# A Rare Case of Urinary Retention Due to Prostate Tuberculosis in Vietnam

**DOI:** 10.7759/cureus.28574

**Published:** 2022-08-30

**Authors:** Hoang Van Hau, Nguyen Van Hieu, Nguyen Dinh Lien, Do Truong Thanh

**Affiliations:** 1 Urology, Hanoi Medical University Hospital, Hanoi, VNM; 2 Urology, Hanoi Medical University, Hanoi, VNM; 3 Internal Medicine, Hanoi Medical University, Hanoi, VNM

**Keywords:** prostate, prostatitis, extrapulmonary tuberculosis, urinary retention, prostate tuberculosis

## Abstract

Prostate tuberculosis is a rare clinical form of extrapulmonary tuberculosis, which causes acute urinary retention infrequently. Cases of prostate tuberculosis reported in the literature often show slow progression with insidious pre-existing urinary disturbances. It can pose a diagnostic dilemma, and the treatment protocols can be challenging. Most clinical cases are managed by antitubercular medications, and surgery is usually reserved for cases where medical treatment fails. Here, we present the case of a 62-year-old male patient who presented to the hospital with acute urinary retention, with a history of dysuria for three months. Ultrasound and magnetic resonance imaging showed enlarged prostate pushing into the bladder lumen with an increasing signal of the parenchyma on T2-weighted imaging. In addition, some lymph nodes were noted near the bilateral iliac vessels pointing toward a chronic inflammation condition with elevated prostatic-specific antigen (4.5 ng/mL). Therefore, transurethral resection of the prostate was carried out for voiding purposes, and the biopsy was sent to the pathology laboratory to rule out malignancy. Intraoperative images revealed a prostatic urethral mass (12 mm × 15 mm × 32 mm) pushing into the bladder lumen, and the histopathological results confirmed the diagnosis of prostate tuberculosis. The patient received a six-month course of antitubercular medications immediately after surgery with a regimen of rifampicin, isoniazid, pyrazinamide, and ethambutol daily for two months, maintaining daily for four months with rifampicin, isoniazid, and ethambutol. Follow-up treatment was conducted two years later, and the results showed a good response with no recurrence. Prostate tuberculosis is best managed with antitubercular chemotherapy, but surgery is unavoidable for acute complications such as urinary obstruction, as seen in this case.

## Introduction

With an estimated prevalence of 322 per 100,000 adult population, tuberculosis is one of the most serious health problems in Vietnam [1]. In addition to pulmonary tuberculosis, the trend of extrapulmonary tuberculosis is also arising, mostly in the brain, spine, joints, and genitals. Prostate tuberculosis is a rare clinical form that accounts for about 2.6% of all extrapulmonary tuberculosis cases [2]. The most common route is the hematogenous spread of tuberculosis to the kidneys and the lower urinary tract subsequently. Clinical symptoms are nonspecific and misleading as other diagnoses such as prostate cancer, prostate hypertrophy, or prostatitis. Although irritative voiding is the most common symptom, only six prostate tuberculosis cases presenting with acute urinary retention have been reported [3]. Here, we report a rare case of urinary retention due to tubercular prostatitis that was found incidentally following transurethral resection.

## Case presentation

**Figure 1:**
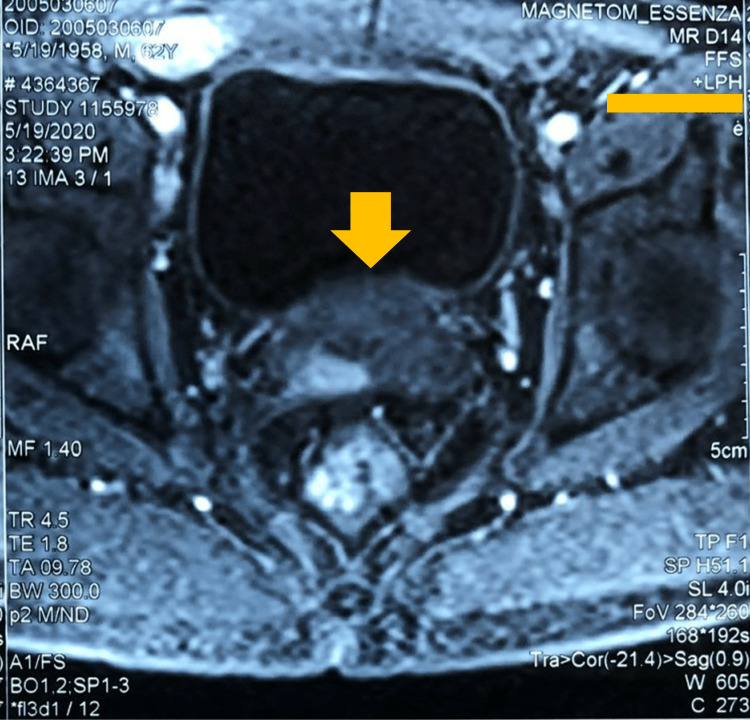
Magnetic resonance imaging showing an enlarged prostate with increasing signal of the parenchyma on T2-weighted imaging.

A 62-year-old man, with a history of dysuria lasting for three months, was admitted to the hospital for acute urinary retention. No history of tuberculosis was recorded. Bladder catheterization was performed with a 22-Fr Foley catheter. In total, 1,200 mL of urine was drained, and the patient became more comfortable. Biological tests revealed a slight increase in prostatic-specific antigen (PSA) level of 4.5 ng/mL and glucose level of 13.4 mmol/L. The human immunodeficiency virus serology was negative, and the urine test showed no infection. Further investigations were performed. The chest X-ray was normal. Abdominal ultrasound revealed an enlarged prostate protruding into the bladder lumen by 12 mm, whose weight was estimated at 80 g; thick bladder wall (about 4.5 mm); and no mass lesions or kidney stones. Magnetic resonance imaging (MRI) revealed numerous lesions in the transitional zone and bilateral peripheral zone of the prostate; an enlarged prostate of 77 g volume with a clear border; the parenchyma of the entire gland elevated slightly with irregular signal on T2-weighted imaging; no early enhancement after contrast injection; and inhomogeneity enhancement in the late phase (Figure 1). The prostate imaging reporting and data system (PIRAD) grading was grade 3. Bilateral iliac vessels had several lymph nodes, the largest measuring 8 × 10 mm.

**Figure 2:**
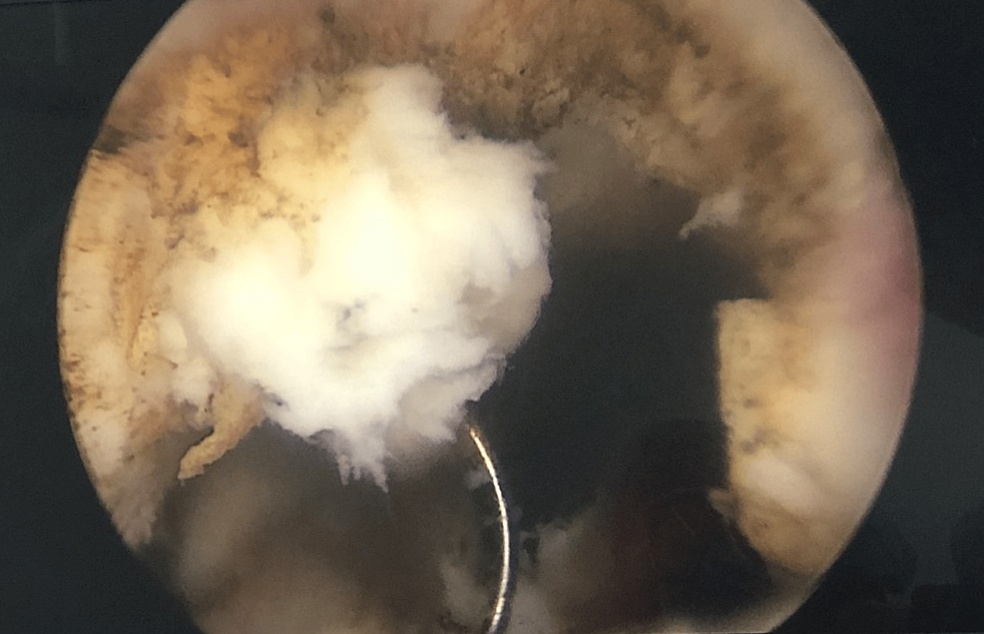
Intraoperative image showing a 12 mm × 15 mm × 32 mm mass pushing into the bladder lumen.

Transurethral resection of the prostate (TURP) was performed to relieve acute compression. Intraoperative image showed a prostatic urethral mass (12 mm × 15 mm × 32 mm) pushing into the bladder lumen, which led to the obstruction (Figure 2).

**Figure 3:**
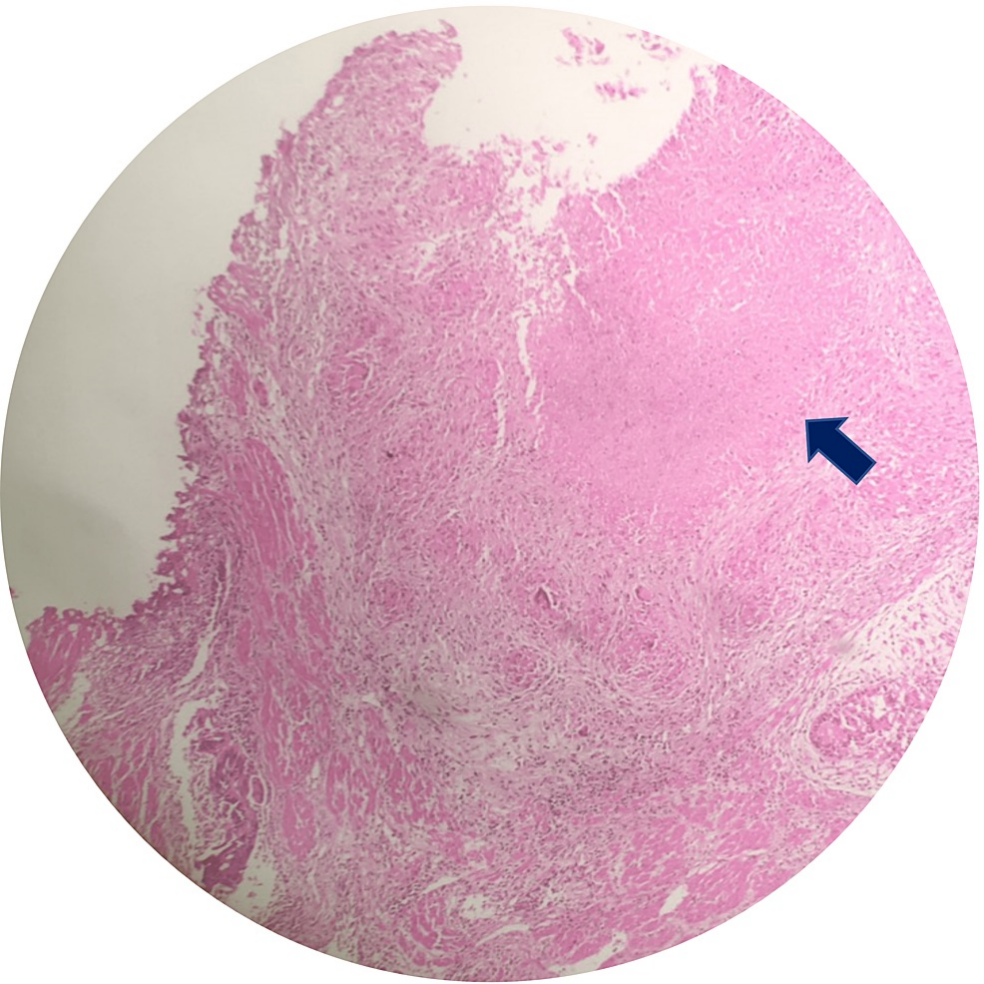
The histopathological examination revealing a typical tuberculous granulomatous lesion. A central caseous necrosis area surrounded by giant cells, mature lymphocytes, transformed lymphocytes, macrophages, plasma cells and fibroblasts, and no malignant cells.

The mass was removed, and the biopsy specimens were collected and analyzed to rule out malignancy. The histopathological result was central caseous necrosis area surrounding by giant cells, mature lymphocytes, transformed lymphocytes, macrophages, plasma cells and fibroblasts, and no malignant cells, which was consistent with tuberculous granulomatous lesions (Figure 3).

The tuberculosis polymerase chain reaction (PCR) test of the urine sample was negative. With characteristic histopathological evidence in favor of tuberculosis, the patient was diagnosed as prostate tuberculosis and was given chemotherapy antituberculosis treatment. The urinary catheter was removed five days later. The urinary retention resolved, and the patient was discharged after seven days. Thereafter, he received outpatient treatment with the regimen of rifampicin, isoniazid, pyrazinamide, and ethambutol daily for two months, along with rifampicin, isoniazid, and ethambutol daily for four months. He was followed up for two years. His PSA level came down to the normal range, and no recurrence was detected.

## Discussion

Prostate tuberculosis is a rare disease of the urinary system with approximately 40 cases reported to date [3]. The majority of cases of prostate tuberculosis are secondary to infections in the lungs, kidneys, bladder, or epidermis [4,5]. There have been numerous clinical cases of primary prostate tuberculosis reported in the literature. For instance, Liang et al. reported a case of prostatic tuberculosis co-occurring with *Escherichia coli* infection in an acquired immunodeficiency syndrome patient [6]. Another primary infection case was reported by López Barón et al. who described a tuberculosis lesion with the normal size of the prostate in a non-immunocompromised patient [5]. In addition, no abscesses but many peripheral lymph nodes are found in MRI, suggesting a chronic inflammatory condition.

Our case may have been a primary prostatic tuberculosis lesion, presenting with lower urinary tract symptoms and acute urethral obstruction. Symptoms including acute urinary retention, slightly raised PSA level, and an enlarged prostate are consistent with prostatic hypertrophy. An inflammatory state of the prostate is shown with the presence of surrounding lymph nodes, with an increasing signal of the parenchyma on T2-weighted imaging of the entire gland on MRI, corresponding with the report by Cheng et al. [7]. According to the authors, prostate tuberculosis often occurs secondarily via the hematogenous route [3]. Immunoassays and PCR tests have high sensitivity of 80% and 95%, respectively, for the diagnosis of tuberculosis [3]. Unfortunately, these new techniques are difficult to access in developing countries like ours. The confirmatory diagnosis was made based on histopathological examination with the appearance of cellular granuloma surrounding central caseous necrosis, which demonstrated specific granulomatous manifestations of tuberculosis.

Prostate tuberculosis lesions often respond very well to antitubercular drugs. According to recent statistics, only a few cases causing urinary retention are reported to be catheterized and treated with antitubercular treatment [3]. Most cases are indicated for surgery when medical treatment with antitubercular drugs has failed. However, surgery is unquestionable for acute complications such as obstruction and should be considered at the onset. In our case, the patient had acute urinary tract obstruction mechanically, and the size of the prostate was medium (80 g); therefore, we decided to perform transurethral resection of the prostate instantly for voiding purposes, and the biopsy was sent to the pathology laboratory to make a diagnosis and exclude malignancy. Finally, the patient was diagnosed with prostate tuberculosis and received appropriate treatment with a good response.

## Conclusions

Primary prostatic tuberculosis is a rare disease of the prostate gland with silent symptoms. It is easily misdiagnosed as prostate cancer, prostate hypertrophy, and prostatitis. Imaging on MRI or CT scans combined with histopathology can help guide accurate diagnosis. The disease is best managed with antitubercular chemotherapy, but surgery is inevitable for acute complications such as obstruction.
